# Neonatal Outcome and Treatment Perspectives of Preterm Infants at the Border of Viability

**DOI:** 10.3390/children9030313

**Published:** 2022-02-24

**Authors:** Rahel Schuler, Ivonne Bedei, Frank Oehmke, Klaus-Peter Zimmer, Harald Ehrhardt

**Affiliations:** 1 Department of General Pediatrics and Neonatology, Justus-Liebig-University, Feulgenstrasse 12, 35392 Giessen, Germany; klaus-peter.zimmer@paediat.med.uni-giessen.de (K.-P.Z.); harald.ehrhardt@paediat.med.uni-giessen.de (H.E.); 2 Department of Obstetrics and Gynecology, Justus-Liebig-University, Klinikstrasse 33, 35392 Giessen, Germany; ivonne.bedei@gyn.med.uni-giessen.de (I.B.); frank.oehmke@gyn.med.uni-giessen.de (F.O.)

**Keywords:** extremely preterm infant, border of viability, decision-making, outcome measures, survival, survival without disability, quality of life

## Abstract

Decision-making at the border of viability remains challenging for the expectant parents and the medical team. The preterm infant is dependent on others making the decision that will impact them for a lifetime in hopefully their best interest. Besides survival and survival without neurodevelopmental impairment, other relevant outcome measures, such as the quality of life of former preterm infants and the impact on family life, need to be integrated into prenatal counselling. Recommendations and national guidelines continue to rely on arbitrarily set gestational age limits at which treatment is not recommended, can be considered and it is recommended. These guidelines neglect other individual prognostic outcome factors like antenatal steroids, birth weight and gender. Besides individual factors, centre-specific factors like perinatal treatment intensity and the attitude of healthcare professionals significantly determine the futures of these infants at the border of viability. A more comprehensive approach regarding treatment recommendations and relevant outcome measures is necessary.

## 1. Introduction

The prognosis of extremely preterm infants has dramatically improved during recent decades. Still today, the implementations of antenatal steroids and postnatal surfactant therapy are listed as the decisive milestones for this positive development. However, it needs to be acknowledged that plenty of further improvements, including respiratory management, better nutritional support, and nosocomial infection prevention programs, among others, have contributed to the better outcomes of extremely preterm infants. In this context, neonatologists around the world have taken into account not only the survival of preterm infants, but also their long-term health status and the long-term sequelae of all organs affected by premature delivery. In many countries of the industrialised world, guidelines and therapy recommendations refer to what is considered the border of viability when specific recommendations are made whether a preterm infant should be actively treated or not. The border of viability has changed over recent decades, and is continuously shifting towards lower gestational ages. Sweden and Japan are among the countries at the forefront of providing care for the most immature extremely preterm infants [1,2]. The legal definition of a viable foetus in the 1960s and 1970s in Sweden was 28 weeks, and therefore the practical rule was not to provide care for infants born <28 weeks [1]. Since that time, the border of viability has decreased stepwise, and is currently considered to be at 22–23 weeks of gestation [1]. Similarly, in Japan, the limit of viability changed from 28 to 24 completed weeks in 1977, and in 1991, from 24 to 22 completed weeks [2]. In contrast, the treatment of preterm infants below 24 weeks of gestation was practically not offered until 2011 in France. Today the “grey zone” area where treatment can be provided in France is still considered from 24 weeks to 25 + 6/7 weeks, but individual centres offer treatment from 22 weeks on [3]. According to a consensus statement in 2017 in the USA, obstetric interventions for threatened or imminent periviable birth are not recommended at 22 weeks of gestation, considered at 23 weeks, and recommended from 24 weeks onward [4]. Generally, national recommendations regarding treatment thresholds at the border of viability are scarce in many parts of the world. The example from India shows that the situation in an upcoming country is even more complex. In the absence of a national consensus and a broad range of medical services ranging from poor healthcare to the best in the world, with a cultural melting pot and multiple religions and cultural backgrounds, decisions on the border of viability are even more difficult [5]. It has become clear that wide variations continue to exist between countries and even within countries as to what is considered the border of viability, and at which gestational age that care of the preterm infant should be offered [6,7,8,9]. In some countries and regions, the treatment of infants of 22 to 23 weeks of gestation is considered the standard of care; in others, it is an exception [2,7,10,11].

Currently, new treatment options such as artificial wombs are studied in premature lambs [12]. Therefore, it is likely that infants considered as non-viable or below the limit of viability today due to physiological limitations, especially regarding lung development, will have a chance of survival in the years to come.

When the treatment outcomes of preterm infants are considered, data are mostly available for survival and survival without neurodevelopmental disability. Generally, survival and survival without NDI has improved for extremely preterm infants over the last two decades, but for those born at 22–23 weeks, this is not universally true. Data from the Neonatal Research Network (USA) from 2000 to 2011 show an improvement of survival and survival without the NDI of infants born at 23–24 weeks, although for the group of infants born at 22 weeks, there was no improvement, and survival was only 2–5%; for infants at 23 weeks’ gestation, survival without neurodevelopmental impairment (NDI) increased from 7% to 13% [13]. In Australia, survival improved from 2009 to 2017 for infants born at 24 weeks, but not for those born at 22 and 23 weeks [14].

Improvements in survival could also be achieved in those infants born at 22–23 weeks of gestation in Sweden. The one-year survival of all live born infants significantly increased from 10% to 30% for those born at 22 weeks of gestation, and from 52% to 61% for those born at 23 weeks of gestation, although not statistically significant [15]. Overall there is a trend towards higher survival rates for infants born at 22–24 weeks of gestation, but differences between countries and within countries remain. One might fear that this increase in survival at the border of viability is at the cost of an increase in infants with severe neurological impairment. Several studies have shown that this is not true, and that an increased survival in this group of infants does not lead to an increase in survival with severe NDI [16,17]. Interestingly, even outcomes for those at later gestational ages are better in countries with a more active approach at lower gestational ages [6].

Improvements in survival and in survival free of major disabilities of extremely preterm infants have led to an adjustment in national guidelines in some countries, for example, in the Dutch guidelines, the threshold for active treatment was lowered from 26 + 0/7 to 25 + 0/7 in 2006 [11].

Most national guidelines for the treatment of preterm infants at the border of viability focus on gestational age limits, where treatment should be provided, should not be provided, and can be provided. In the “grey zone” area where treatment can be provided, it is a joint decision of the parents together with the medical team [1,7]. The person at the centre of the decision cannot voice her or his opinion, and therefore is dependent on others making decisions in hopefully their best interest. Gestational age at birth survival and whether the child will be disabled are often the focus of guidelines and prenatal counselling. It is questionable whether these are comprehensive criteria for this decision.

Other factors beyond gestational age impact the prognosis, including baseline factors, such as antenatal steroids [18,19], gender [20,21], birth weight [21,22], possibly socioeconomic status [23] and factors not attributable to the individual case but the centre practice [24] and attitudes [25].

The application of antenatal steroids is known to improve preterm outcomes. In a recent European cohort study of the EPICE (Effective Perinatal Intensive Care in Europe) network antenatal steroids given even shortly before preterm birth reduced the risk of death or severe brain damage [18]. For antenatal steroids, the latest large cohort study inter alia from the USA demonstrates a survival advantage, even when initiated at 22 completed weeks of gestation [19]. This shows that backbones of therapy, such as antenatal steroids, need a careful evaluation before a lack of efficiency should be stated below a certain limit of gestational age.

Other individual factors, such as female sex, increased birth weight and singleton birth, were associated with reductions in risk of death and neurodevelopmental impairment. [21]. Therefore, better risk calculation beyond gestational age is necessary to give a fair prognosis estimate. One such approach is the NICHD risk calculator (https://www.nichd.nih.gov/research/supported/EPBO/use; accessed on 21 February 2022), while one shortcoming is that only weeks and not days can be entered, and the variation in outcome between “early” versus “late” 23 weeks has been described [26].

## 2. Outcome and Outcome Determining Factors

### 2.1. Gestational Age

Gestational age determined by last menstrual period or ultrasound is known to be imprecise. Dating by last menstrual period, which is common in many parts of the world if ultrasound is not available, is prone to imprecision, as the accurate recollection of this date cannot be assumed. A first trimester ultrasound provides an estimate of gestational age, but errors of up to 7 days have been described [4,27,28].

Based on the scientific data, it needs to be acknowledged that the borders of viability are set arbitrarily and cannot reflect the gradual increases in outcome prognosis, as survival and disability-free survival increase steadily with increasing gestational age [8]. This seems to be true for every one-day increment in gestational age throughout extreme prematurity [22], but remains true even up to term age [29]. Fixed borders, as it is current practice in national guidelines and recommendations whether a preterm infant should be resuscitated or not do not meet the individual situation. There is no greater difference between the outcomes of an infant born at 23 + 6/7 compared to 24 + 0/7 and between the outcomes of an infant at 24 + 5/7 compared to an infant at 24 + 6/7 weeks, but if the treatment threshold is at 24 + 0, one day will decide between life and death in the first scenario.

Although gestational age does influence these relevant outcome measures and of course has to be considered when treatment options are discussed, the variability of survival and disability free survival is considerable. Survival rates of preterm infants before 28 weeks vary between 0% [14] and 70% [30] at 22 weeks, and between 89% and 97% at 28 weeks [6].

Birth weight [22], neonatal morbidities [31], the region or country of birth [6,13,32], pre- and postnatal treatment intensity [24] and the attitude and expectations of the medical team [25,33] all influence survival and survival without NDI, explaining the substantial differences in outcome at the same gestational age.

### 2.2. Neonatal Morbidities

Bronchopulmonary dysplasia (BPD), retinopathy of prematurity (ROP) and serious brain injury (e.g., intraventricular haemorrhage) are neonatal morbidities with a significant negative influence on the neurodevelopmental outcome of preterm infants. Each additional morbidity aggravates the odds of poor outcome. For very low birthweight infants surviving without any morbidity, the probability of a poor outcome at 5 years of age was only 11% [31]. In several cohort studies, morbidities involving inflammatory processes are associated with adverse neurological outcomes. [34,35,36,37] Preterm infants with necrotizing enterocolitis have lower IQ results at 6 years of age, and those with late-onset bacteremia have poorer neurocognitive outcomes at 10 years of age [36,37]. As neonatal morbidities are such a great influencing factor for the outcome of preterm infants, it is important to discuss the postnatal redirection of care with expectant parents as an option if their child has one or more neonatal morbidities impacting the expected neurodevelopmental outcome. As neonatal morbidities obviously cannot be integrated into risk assessment and expected outcome prenatally, it is important to address the redirection of care as an option postnatally.

### 2.3. Outcome Measures

Obviously, over the lifetime of a former preterm infant, more outcome measures than survival and survival without NDI are relevant. Therefore, several birth cohorts have been followed into later childhood, adolescence, and adulthood. Overall, academic achievement for preterm infants is lower than for their term counterparts [38]. In a population-based cohort of preterm infants in the UK and Ireland (EPICure), almost half had moderate to severe impairment in reading and maths at the age of 11 years, but 87% were attending mainstream school [39]. More important than academic achievement is quality of life (QoL) and social functioning, as these are the factors that determine the personal situation and wellbeing of former preterm infants.

In most studies, social functioning is assessed with indicators, such as educational attainment, income level, being employed or being in a romantic relationship. With this approach, former preterm infants often have lower levels of social functioning [40,41]. How former preterm infants perceive their experiences and relationships with others is the focus of a recent individual participant data meta-analysis. This approach assesses social function in a much more relevant and meaningful way for the individual. Interestingly, former very preterm/very low birthweight (VP/VLBW) adults perceived their relations with partners and family and their experience of work and education as similar to those in the former term control group, while they had lower ratings in their relationships with friends [42]. The self-reported mental health was comparable between former preterm and term infants [43]. A study from Switzerland showed no difference in the health related quality of life (HRQoL) of former preterm infants compared to term counterparts, even though they did tend to have more chronic diseases [44]. Having a biological impairment therefore does not necessarily mean that the individual has a poor self-assessed QoL [45]. An Australian cohort study yielded similar results, with no difference in QoL between former extremely preterm infants and term counterparts [46], whereas a cohort study from France [47] found QoL to be poorer for those born as extremely preterm infants. Interestingly, in those studies where parents function as proxy respondents for the former preterm infant, they judge the QoL differently [47], and often lower than the individual whose QoL is being judged [45]. QoL can only be described by those living the life, and cannot be determined through objective outcome measures or the view of others on that life [33].

One of the challenges in predicting longer-term outcome is that current preterm infants in adulthood received a very different neonatal care, as medical treatment has greatly advanced during the last 10–20 years; therefore, outcome is possibly even better. On the other hand, preterm infants today are treated at much lower gestational ages than several years ago, with unknown longer-term consequences. Currently, no cohort data on QoL in adolescence of those born at 22–23 weeks of gestation exist. Being aware of these uncertainties during counselling and decision-making and interpreting data cautiously is important [33].

### 2.4. Family Situation

The outcome for the individual preterm infant has to be at the centre of the decision regarding treatment at the border of viability. However, the family as a whole will greatly be affected, and therefore also needs to be considered. Some parents worry about the possible burden that the preterm infant will place on their marriage and family life. Maternal depression scores are higher in mothers of preterm infants compared to those of term infants postnatally, whereas at three months, this difference disappears [48]. The impact of preterm birth on families at adolescence was evaluated regarding family functioning in a birth cohort of 1977–1982, compared to a term control group. Overall, the families of former preterm infants had adapted well, and there were no differences in the two groups regarding emotional suffering or worry of parents because of their child’s physical or emotional health, or regarding available time for personal needs. Parents of former extremely low birthweight infants (ELBW) infants did feel that their child’s health had an impact on their own emotional health, alongside other negative effects on the family. The impact on marriage was mixed, preterm birth was a major factor in separation and divorce, parents of ELBW infants reported more stresses and strains on their marriage, but also that it had brought the partners and family closer together, family and friends were more understanding, and that the experience improved their feelings about themselves. Interestingly, more parents of disabled ELBW infants reported improved feelings about themselves [49]. At young adult age, there was no difference on marital disharmony, family dysfunction, social support scores and maternal depression and anxiety between families of former ELBW infants and term infants. Paradoxically, mothers of young adults with neurosensory impairment (NSI) reported significantly less family dysfunction compared to those without NSI. Mothers in the ELBW group did report negative effects on their and their spouse’s work [50]. Although the emotional wellbeing of the family of an ELBW infant is negatively affected in the beginning, over the following years, the positive effects increase, and ultimately maybe even outweigh the negative ones.

Parental education and socioeconomic status have been described as risk factors for the poorer neurodevelopmental outcome of preterm infants. In a recent study, parental educational level did not modify the relationship between gestational age and educational attainment in adulthood, although across all gestational age groups up to term age, lower parental education level was associated with lower educational attainment at 25 years [23].

### 2.5. Regional Differences

The International Network for Evaluating Outcomes of Neonates (iNeo) compared outcome data of 10 national and regional neonatal networks of infants born at 24–29 weeks of gestational age. Overall survival rates varied from 78% to 87%, with the greatest difference for infants born at the lower gestational ages. The difference at 24 weeks’ gestation was 35% versus 84% survival, and followed an identical pattern for those born at 25–29 weeks of gestation, but with less pronounced differences [6]. Besides differences in outcome between countries, these also exist within countries. In a large Swedish cohort study including all births at 22–26 weeks’ gestational age from 2004 to 2007 (EXPRESS), the perinatal mortality rate ranged from 22% to 46%, and the regional variations in mortality were wide for those born at 22–24 weeks gestation, while not different at 25–26 weeks gestation [32]. A large cohort study of the Eunice Kennedy Shriver National Institute of Child Health and Human Development Neonatal Research Network (NICHD) yielded similar results of significant inter-hospital variations in the USA [51]. In the updated NICHD outcome model, hospital of birth contributed equally as much as gestational age to the estimation of survival [20]. Single centres with a uniformly active or very active approach have survival rates of 50–70%, even for infants at 22 weeks’ gestation, and those surviving around half of that have no or mild neurodevelopmental impairment at 18–30 months corrected age [10]. Results from references [30,52] can be interpreted as the current best possible outcome if all efforts are made towards saving the life of these most vulnerable infants.

### 2.6. Intensity of Perinatal Support

Several studies have shown that more active perinatal support leads to increased survival rates [24,30,51]. Crucial factors for an active perinatal approach are both obstetrical and neonatal. Obstetrical interventions considered as indicators for an active approach are the following: delivery at a level III hospital, complete course of antenatal steroids, caesarean delivery, and tocolytic treatment. In a large population-based prospective cohort study in Europe (EPICE), the administration of corticosteroids was estimated to have reduced the neonatal mortality by 51%, and significantly reduced neonatal morbidities [18]. Neonatal indicators are surfactant administration, delivery attended by a neonatologist, intubation or application of continuous positive airway pressure (CPAP) immediately after birth, and admission to intensive care [24]. In the Swedish EXPRESS study infants born in centres with an active perinatal approach at 22–24 weeks had a risk of 62.3% of death or NDI at 2.5 years corrected age, compared to a risk of 79.3% in the centres with less perinatal activity. Major neonatal morbidities and any NDI were lower in the group treated more actively, although not statistically significant. Therefore, the active treatment of these infants born at the border of viability does not only lead to improved survival at the cost of more infants with NDI, but neurodevelopment is even better [53].

### 2.7. Attitude of Healthcare Professionals

The relative value of a preterm life is often viewed as less than that of older children. In a questionnaire-based study, physicians and university students were investigated regarding the likelihood of resuscitation of different patients with the same outcome regarding survival and survival with or without neurological impairment. The just-delivered 24-week preterm infant was the least likely to be resuscitated, although the chance of survival and survival without disability was the same as that of a 2-month-old with meningitis, a baby just born with a known malformation, and a 50-year-old in a car accident [25]. Attitudes between obstetricians and neonatologists regarding the treatment of infants born at the border of viability differ: one study shows a more proactive approach at lower gestational ages of neonatologists [54], another study shows the opposite with obstetric caregivers more likely to resuscitate infants at 22–23 weeks’ gestation [55]. Colleagues in Brazil investigated what influence disagreement regarding the treatment of preterm infants has on neonatal outcome. They found an increased relative risk of death (2.45; 95% CI 1.16 to 3.47) within 24 h if doctors disagreed on whether the infant should be treated or not [54]. This emphasizes that the attitude of the whole team is important, as suboptimal prenatal treatment will result in worse outcomes, in spite of proactive postnatal treatment [54]. Positive and negative attitudes will result in a self- fulfilling prophesy, which is most evident if no treatment is offered at 22 weeks’ gestation. Without resuscitation, no infant will survive, which reinforces the original recommendation [33].On the positive side, centres with a favourable outcome at 22–23 weeks of gestation are much more likely to offer treatment with continuously good results [10].

## 3. Conclusions and Recommendation

It becomes evident from our review and the available extensive scientific data on the topic that, more than gestational age, survival and survival without NDI need to be considered for decision-making at the border of viability.

Clearly, the outcome has changed over the last decade, especially among those born at 22–24 weeks of gestation, leading to the adaptation of guidelines in several countries [11]. However, decision criteria in these guidelines have basically remained the same, with a continued focus on gestational age as a limit for intervention. Alongside the imprecision of gestational age, the documented substantial differences in mortality and short- and long-term outcomes at the same gestational age show that basing intervention thresholds mainly on gestational age is arbitrary and highly controversial [52].

If the decision for treatment has been taken, it is important to actively manage the perinatal period, as this yields the best results. Redirection of care should always be an option if severe complications occur, medical treatment becomes futile [56], and the burdens of treatment and prolongation of life support are not justified by the expected quality of life.

Survival without neurodevelopmental impairment does not equal a good quality of life. Moreover, although preterm infants cannot be asked at the beginning of their life whether they wish for treatment or not, we can consider the judgement of those who were preterm 10–20 years ago regarding outcomes like quality of life in adolescence and adulthood. Obviously, shorter-term outcomes (neonatal morbidities, neurodevelopment) can be assessed timelier, and will remain important prognostic factors, but they need to be interpreted cautiously and in light of a whole lifetime. Possibly the most important influenceable factor is the attitude of each individual caring for these most vulnerable patients and their families. If the care for these infants is done with utmost perinatal intensity and with a positive attitude, outcomes of preterm infants at the border of viability will continue to improve, regardless of where the border is seen within the physiologic limits of nature.

## Data Availability

Not applicable.
